# Rainbow of KIBOU (ROK) study: a Breast Cancer Survivor Cohort in Japan

**DOI:** 10.1007/s12282-017-0784-x

**Published:** 2017-05-25

**Authors:** Yuri Mizota, Yasuo Ohashi, Takuji Iwase, Hiroji Iwata, Masataka Sawaki, Takayuki Kinoshita, Naruto Taira, Hirofumi Mukai, Seiichiro Yamamoto

**Affiliations:** 1 Division of Health Sociology, Center for Public Health Sciences, National Cancer Center, 5-1-1 Tsukiji, Chuo, Tokyo, 104-0045 Japan; 2 Integrated Science and Engineering for Sustainable Society, Chuo University, 1-13-27 Kasuga, Bunkyo, Tokyo, 112-8511 Japan; 3 Breast Oncology Center, The Cancer Institute Hospital of Japanese Foundation For Cancer Research, 3-8-31 Ariake, Koto, Tokyo, 135-8550 Japan; 4 Department of Breast Oncology, Aichi Cancer Center Hospital, 1-1 Kanokoden, Chikusa-ku, Nagoya, Aichi 464-8681 Japan; 5 Department of Breast Surgery, National Cancer Center Hospital, 5-1-1 Tsukiji, Chuo, Tokyo, 104-0045 Japan; 6 Department of Breast and Endocrine Surgery, Okayama University Hospital, 2-5-1 Shikata-cho, Kita-ku, Okayama, Okayama 700-8558 Japan; 7 Department of Breast and Medical Oncology, National Cancer Center Hospital East, 6-5-1 Kashiwanoha, Kashiwa, Chiba 277-8577 Japan

**Keywords:** Breast cancer, Patient cohort, Lifestyle, Psychosocial factor, Survivorship

## Abstract

**Background:**

Although there are a large number of epidemiological studies investigating the etiological role of lifestyle factors in breast cancer, there are few studies on the association between lifestyle factors and breast cancer prognosis. To investigate the influence of lifestyle factors such as diet and physical activity, use of complementary and alternative medicine, and psychosocial factors on prognosis, we designed a large-scale cohort study of female breast cancer patients in Japan.

**Methods:**

The planned sample size is 7200. The cohort is being conducted in collaboration with several clinical trials, a cancer registry, and daily practice. Information on clinical factors, treatment, and follow-up will be obtained from the clinical trials and participating hospitals. A self-administered questionnaire is given to subjects before, immediately after, or 1 to 5 years after surgery. Blood and tissue samples are also collected. The primary endpoint is disease-free survival. The secondary endpoints are overall survival and health-related quality of life. The follow-up period will be at least 5 years after the last participant is enrolled. Recruitment began in November 2007.

**Current status:**

As of April 2017, there are 5852 patients enrolled in the study along with 1430 biological samples and the study is still ongoing. The number of subjects enrolled in the study is already the largest in the world.

**Conclusions:**

The ROK study will provide much important evidence for breast cancer survivorship.

## Introduction

There is a growing number of cancer survivors in the world. Especially, since the prognosis of breast cancer is relatively favorable compared to other types of cancer [1], there are a large number of breast cancer survivors. In addition to medical treatments, patients are also willing to make their own efforts to prevent recurrence, such as changing their diet and levels of physical activity and incorporating complementary and alternative medicine (CAM) into their daily lives. Although there is growing number of epidemiological studies investigating the etiological role of lifestyle factors in breast cancer, many fewer studies have examined the association between modifiable risk factors such as lifestyle and CAM use and breast cancer recurrence and survival [2, 3]. Recently, several prospective cohort studies have been designed and are being conducted [4, 5], but as the studies have just started, sufficient evidence has not been obtained. Such information is eagerly awaited by breast cancer patients as well as breast cancer researchers.

In addition to these factors, psychosocial factors play important roles on patient’s quality of life (QOL). For this purpose, the association between lifestyle and psychosocial factors and prognosis should be studied, and appropriate information must be provided to patients, their families, and health professionals.

In this ROK (Rainbow of KIBOU) study, a large cohort of breast cancer patients is being conducted to examine the influence of lifestyle factors, CAM use, psychosocial factors, pain, and supportive care on prognosis such as QOL, recurrence, and survival. Furthermore, in part of the cohort, blood samples and tissues are being collected to examine the association between prognosis and several biomarkers such as serum nutrient levels and genetic polymorphisms, and somatic mutations.

## Methods

### Study structure

The ROK study consists of several cohorts (Table 1). Some of the cohorts are designed as part of a collaborative study with several multi-institutional randomized control trials involving female breast cancer patients. A self-administered questionnaire is delivered to patients enrolled in these trials, and their responses are regarded as baseline data of the cohort. Follow-up and clinical information will be obtained from the participating institutions. We are conducting 3 cohorts in collaboration with clinical trials: cohort 05, cohort 06, and cohort 07. Cohort 05 is a collaborative study with the National Surgical Adjuvant Study of Breast Cancer (N-SAS BC) 05; Randomized Study to Assess the Efficacy of a Further 5 Years of Anastrozole Treatment for Postmenopausal Women with Breast Cancer Completing 5 Years of Anastrozole Containing Adjuvant Endocrine Therapy (AERAS). Cohort 06 is a collaborative study with the N-SAS BC06; Randomized Phase III Study of Adjuvant Endocrine Therapy with or without Chemotherapy for Postmenopausal Breast Cancer Patients who Responded to Neoadjuvant Letrozole (NEOS). Cohort 07 is a collaborative study with N-SAS BC07; Evaluation of Trastuzumab without Chemotherapy as a Postoperative Adjuvant Therapy in HER2-positive Elderly Breast Cancer Patients: Randomized Controlled Trial (RESPECT). These trials are directed by the Comprehensive Support Project for Oncology Research (CSPOR) of the Public Health Research Foundation, Tokyo, Japan.

**Table 1:** Outline of the subcohorts of the Rainbow of KIBOU study

Subcohort	Cohort outline	Enrolment	Current status (as of April 30, 2017)
Cohort 05	Collaboration with N-SAS BC05 (Randomized Phase III Study to Assess the Efficacy of a Further 5 Years of Anastrozole Treatment for Postmenopausal Women)	Nov 2007–Mar 2014	1510 patients enrolled from 99 institutions (response rate: 94.8%)
Cohort 06	Collaboration with N-SAS BC06 (Randomized Phase III Study of Adjuvant Endocrine Therapy with or without Chemotherapy for Postmenopausal Breast Cancer Patients who Responded to Neoadjuvant)	May 2008–Sep 2013	735 patients enrolled from 78 institutions (response rate: 95.8%)
Cohort 07	Collaboration with N-SAS BC07 (Evaluation of Trastuzumab without Chemotherapy as a Postoperative Adjuvant Therapy in HER2 Positive Elderly Breast Cancer Patients: Randomized Controlled Trial)	Oct 2009–Sep 2015	311 patients enrolled from 85 institutions (response rate: 95.4%)
Cohort NCC	Single institution study (All breast cancer patients treated at National Cancer Center Hospital)	Nov 2011-	1592 patients enrolled with biological samples and 1236 questionnaires
Cohort Setouchi	Collaboration with Setouchi Breast Cancer Cohort Study conducted by Nonprofit Organization Setouchi Breast Project Comprehensive Support Organization	Feb 2013-	1704 patients enrolled from 15 institutions with 1496 questionnaires
Total			5852 patients (7200 planned)

In addition to the cohorts associated with these clinical trials, other cohorts are being recruited at the National Cancer Center Hospital in Tokyo, Japan (cohort NCC) and a collaborative study with the Setouchi Cancer Registry (cohort Setouchi). Blood and tissue samples are being collected in cohort NCC in addition to questionnaire data.

### Participants of the study

Eligibility criteria for cohorts 05, 06, and 07 are based on the eligibility of the respective clinical trial. Patients who give informed consent for participation in the trials are considered eligible for the cohort study. The eligible subjects for the N-SAS BC05 study are postmenopausal women with hormone-responsive primary breast cancer aged less than 80 years. The eligible subjects for the N-SAS BC06 study are postmenopausal women with primary breast cancer aged less than 75 years [6]. The eligible subjects for the N-SAS BC07 study are women with HER2-positive primary breast cancer aged between 70 and 80 years [7]. Details are available at http://www.umin.ac.jp/ctr/ (Protocol ID: UMIN000000818 for N-SAS BC05, UMIN000001090 for N-SAS BC06, and UMIN000002349 for N-SAS BC07). For cohorts NCC and Setouchi, the eligibility criteria include females with primary breast cancer, age 20 years or older, anticipating surgery at the National Cancer Center Hospital or institutions participating in the Setouchi Cancer Registry. Among patients meeting the eligibility criteria, those who provide informed consent for participation in this study are considered eligible.

### Survey items and measurements

Questions regarding lifestyle factors are based on the questionnaire used in the Japan Public Health Center-based Prospective Study (JPHC study) [8]. In Europe and the United States, cohort studies have been conducted to evaluate the influence of lifestyle factors such as diet and physical activity, as well as obesity, on recurrence in breast cancer patients [2, 3]. Unfortunately, there are still very few such studies, and sufficient evidence has not been obtained.

In addition to diet and exercise, many patients are interested in and actively practice CAM. This includes the use of oral and topical agents and health-promoting methods such as acupuncture, moxibustion, and yoga, which differ from health insurance-supported practice. Although approximately 50% of cancer patients utilize CAM in Japan [9], there is insufficient evidence regarding its efficacy [10]. CAM utilization is assessed with items based on previous studies [9].

Psychosocial issues reported in breast cancer patients include depression and hopelessness, escape-avoidance coping styles, and stress related to socioeconomic changes [11, 12]. Some previous studies have reported an association between hopelessness, coping style, and recurrence, whereas others have found no association [13, 14]. Therefore, in clinical practice, strategies to manage these psychosocial issues tend to be actively pursued. To evaluate psychological wellbeing, we employ the Herth Hope Index (HHI) [15]. To assess depression, the Center for Epidemiologic Studies Depression Scale (CES-D) [16] is used.

In cohort NCC, blood is collected from the participant. Using plasma or serum samples, the following is the list of candidate substances to be measured: endogenous hormones, insulin and insulin-like growth factor, adipocytokines, markers of inflammation, nutrients, and other substances.

The following is the list of candidates genes to be assessed using DNA samples: genetic polymorphisms in hormone synthesized and metabolized genes, hormone receptor genes, genes related to insulin-like growth factor, genes related to one carbon metabolism, vitamin D receptor genes, genes related to oxidative stress, and genes related to the metabolism of therapeutic agents.

### Endpoint

The primary endpoint is disease-free survival. The secondary endpoints are overall survival and health-related quality of life (HRQOL). HRQOL is measured using the Medical Outcome Study-Short Form 36 (SF-36) [17], EuroQol 5 Dimensions [18], Hospital Anxiety and Depression Scale [19], CES-D, Functional Assessment of Cancer Therapy(FACT)-General [20], FACT-Breast [21], FACT-Endocrine Symptoms [22], FACT-Taxane [23], Patient Neurotoxicity Questionnaire [24], Mini-Mental State Examination [25], and Philadelphia Geriatric Center Moral Scale [26].

### Data collection

**Fig. 1:**
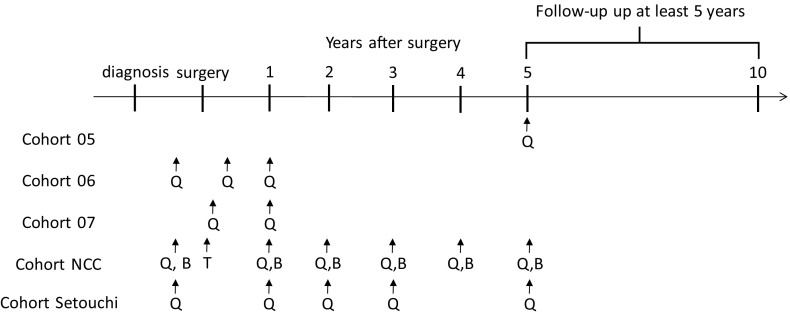
Survey timing of Rainbow of KIBOU study. *Q* questionnaire, *B* blood sampling, *T* tissue sampling

The timing of survey administration during the disease course is shown in Fig. 1. For cohorts 05, 06, and 07, upon registration in the clinical trial, attending physicians explained the purpose and contents of this study, and handed out a self-administered questionnaire to the subjects. They completed the questionnaire at home and returned it by mail. The questionnaire survey was administered once to each participant in cohort 05. In cohort 06, the questionnaire was administered upon primary registration into the clinical trial before surgery, within 8 weeks after surgery, and 12 months after the start of the postoperative treatment protocol for a total of 3 times. In cohort 07, the questionnaire was administered on primary registration into the clinical trial after surgery and 1 year after the start of the postoperative protocol treatment.

In cohorts NCC and Setouchi, the questionnaire is administered upon primary registration into the study before surgery and each year after surgery for 5 years for a total of 5 or 6 times. For each survey, attending physicians explain details of this study, and hand out a questionnaire. In cohort NCC, blood sampling is also conducted at the same time as the questionnaire.

### Sample size and study period

The target number of registered patients is 7200 in total. The cohort 05, 06, and 07 finished their registration and the cohort NCC and Setouchi are still enrolling patients. The follow-up period will last for 5 years after the last participant has been enrolled, except for cohort 07, in which patients will be followed 3 years after the last patient was enrolled. The association between the number of expected events and the hypothesis being investigated is shown in Table 2.

**Table 2:** Number of necessary events and sample size to obtain 80% statistical power

Scenario		Necessary number in two groups^a^	
10-year disease-free survival in unexposed group (%)	Hazard ratio of exposed to unexposed	Events	Sample size
85	0.8	638	6154
85	0.7	254	2590
85	0.6	128	1380
80	0.8	638	1922
80	0.7	254	5678
80	0.6	128	1024
75	0.8	638	3626
75	0.7	254	1522
75	0.6	128	808

^a^Necessary numbers are calculated with scenario where 5-year accrual and 5-year follow-up

### Statistical methods

The association between questionnaire items and subsequent prognosis will be examined. The data from the clinical trials is managed at the CSPOR data center and the questionnaire data is managed at the epidemiologic data center for the cohort study. The effect of factors of interest on outcomes will be examined using regression models that adjust for the effects of potential confounders. The models to be used (Cox, logistic, and linear, etc.) will be chosen based on the type of outcome variable.

Subcohorts will be analyzed separately and also analyzed as a combined cohort. As shown in Fig. 1, several subcohorts have collected data on the same timing. For example, cohort 06, 07, NCC and Setouchi collected lifestyle data before surgery and 1 year after surgery. These data are analyzed as a baseline data compared with prognosis. As for cohort 05, NCC and Setouchi, they collected data 5 years after surgery and these data can be analyzed for the association with the prognosis after 5 years. In principle, as for the combination of all the cohorts with different timing from surgery, we plan to analyze the data with meta-analytic approach. Meta-analytic approach can be justified when the effect of interest are the same across cohorts.

### Ethical approval

A protocol was prepared for each subcohort and approved by the Institutional Review Board of each participating institution. All investigators participating in this study are compliant with the guidelines for research ethics in Japan and the Helsinki Declaration.

## Current status

In total, there are already 5852 subjects enrolled as of April 30, 2017 (Table 2). The registration of cohort 05, 06, 07 successfully ended with 1510, 735, and 311 patients enrolled respectively. The participation rates are surprisingly high with more than 95%. The registration of cohort NCC and Setouchi are still ongoing with already 1592 and 1704 patients enrolled.

Table 3 shows selected baseline characteristics of the ROK study based for already closed cohort 05, 06, and 07. These collaborative cohorts with the clinical trials have a limited age range due to the eligibility criteria of the trials. Patients are enrolled from institutions all over Japan. About 70% of the subjects stated they are “married,” but others answered that they have no husbands for cohort 05 and 06. In cohort 07, the proportion is “married” is much lower since it has a much older age distribution. As for the questions concerning current employment, about 60% of the subjects replied that they are housewives or unemployed in cohorts 05 and 06, and 75% in cohort 07. Regarding CES-D, more than 30% revealed a tendency towards mild (16 ≤ score ≤ 26) or severe depression (score ≥27) when they were asked after breast cancer diagnosis (cohort 06, 07) but the proportion of mild and severe depression was much lower (19%) when CES-D was administered 5 years after diagnosis (cohort 05). More than 95% of each cohort endorsed that they have had at least one perceived positive change after the diagnosis of breast cancer and the number of perceived positive changes tended to be larger in cohort 05, for whom data was collected 5 years after diagnosis. The HHI distribution did not vary substantially across cohorts.

**Table 3:** Baseline characteristics of the ROK study subjects for cohort 05, 06, and 07

	Cohort 05		Cohort 06		Cohort 07	
	5 years after diagnosis (*n* = 1510)		Before surgery (*n* = 755)		After surgery (*n* = 313)	
	*n*	%	*n*	%	*n*	%
Age (years)						
Total	1510	100.0	755	100.0	313	100.0
20–29	0	0.0	0	0.0	0	0.0
30–39	0	0.0	0	0.0	0	0.0
40–49	9	0.6	3	0.4	0	0.0
50–59	365	24.2	197	26.1	0	0.0
60–69	774	51.3	435	57.6	0	0.0
70–79	350	23.2	120	16.0	303	96.8
≧80	12	0.8	0	0.0	10	3.2
Location of institute						
Total	1510	100.0	755	100.0	313	100.0
Hokkaido	100	6.6	73	9.7	25	8.0
Tohoku	43	2.8	86	11.4	22	7.0
Kanto	622	41.2	226	29.9	92	29.4
Chubu	281	18.6	122	16.2	60	19.2
Kinki	178	11.8	111	14.7	55	17.6
Chugoku	143	9.5	64	8.5	18	5.8
Shikoku	25	1.7	4	0.5	7	2.2
Kyushu	118	7.8	69	9.1	34	10.9
Marital status						
Total	1510	100.0	735	100.0	311	100.0
Married	1078	71.4	523	71.2	163	52.4
Divorced	82	5.4	59	8.0	9	2.9
Separated	13	0.9	6	0.8	2	0.6
Widowed	210	13.9	89	12.1	92	29.6
Unmarried	105	7.0	49	6.7	9	2.9
Others	4	0.3	1	0.1	0	0.0
No answer	18	1.2	8	1.1	36	11.6
Current job						
Total	1510	100.0	735	100.0	311	100.0
Self-employed	111	7.4	67	9.1	19	6.1
Full-time employee	146	9.7	91	12.4	2	0.6
Part-time employee	255	16.9	127	17.3	13	4.2
Housewife	703	46.6	315	42.9	160	51.4
Unemployed	227	15.0	98	13.3	75	24.1
No answer	68	4.5	37	5.0	42	13.5
CES-D^a^						
Total	1441	100.0	693	100.0	246	100.0
0–7	439	30.5	163	23.5	53	21.5
8–15	735	51.0	294	42.4	113	45.9
16–26	219	15.2	172	24.8	57	23.2
≧27	48	3.3	64	9.2	23	9.3
Perceived positive change^b^						
Total	1510	100.0	735	100.0	311	100.0
0	32	2.1	36	4.9	12	3.9
1–3	229	15.2	155	21.1	62	19.9
4–6	410	27.2	232	31.6	102	32.8
≧7	839	55.6	312	42.4	135	43.4
Herth Hope Index^c^						
Total	1450	100.0	713	100.0	268	100.0
1–20	7	0.5	9	1.3	5	1.9
21–30	210	14.5	102	14.3	33	12.3
31–40	914	63.0	439	61.6	170	63.4
≧41	319	22.0	163	22.9	60	22.4

^a^Center for Epidemiologic Studies Depression Scale [16] is a self-report depression scale with higher score indicating more psychiatric disorder (total score range 0–60)

^b^Positive changes or gains from experience of breast cancer with higher score indicating more positive change. 9 items such as “I grew as a person,” “family ties became stronger” (total score range 0–9)

^c^Herth Hope Index [15] is a 12 item scale to measure hope with higher score indicating higher level of hope (total score range 12–48)

## Special feature of the ROK study

The ROK study is one of the first comprehensive prospective cohort studies investigating the influence of lifestyle factors, use of CAM, psychosocial factors, pain, and supportive care on prognosis, including QOL, recurrence, and death in patients with breast cancer. The ROK study has already enrolled more than 5800 subjects and is one of the largest breast cancer cohort studies in the world. Participation rates are greater than 90% and approximately 65 participants are recruited each month. Compared with other patients cohort [2–5], the ROK study is the second Asian [27] and the first Japanese cohort and only cohorts who includes long time survivor at least 5 years post-diagnosis. We are planning to enroll patients for another 2 years to achieve the target sample size. Since the follow-up periods of the cohort 05 and the cohort 07 will ends in 2017, the first result can be appeared in near future. The combined results of all the cohorts will also be gradually published.

The strengths of the ROK study include having subcohorts as collaborations with clinical trials. If it were in observational studies, treatment is one of the strongest confounders, but this confounding effect is almost perfectly controlled in randomized controlled trials. In addition, data on other potential confounders related to clinical factors are precisely collected in clinical trials. Recurrence can be used as an endpoint in the ROK study because a valid and reproducible definition is used to confirm the date of recurrence in clinical trials, whereas most observational cohort studies can only use death as an endpoint. Another strength is that many hypotheses can be investigated in the ROK study since it collects data before diagnosis, after surgery, and every year after surgery until the 5th year in the same subcohorts. Since institutions from all over Japan are participating in the study, there is a diverse variability in exposure and generalizable results will be obtained. Since blood and tissue data are collected for cohort NCC, results from the questionnaire data can be validated using biomarkers, for example, for the association between certain nutrients and recurrence. Gene-environment interactions can be investigated and subtype specific analysis based on the gene profile can be also conducted.

In addition to the lifestyle factors, the ROK study focuses on psychosocial factors. One of the main objectives of the ROK study is to investigate the psychosocial factors influencing survivorship, including the HHI, perceived positive change due to cancer, *ikigai* (source of joy and reasons for living), job, social activities, and support that are important to living with breast cancer. This study will clarify the importance of these factors in terms of their association with prognosis and long term QOL. It will provide evidence-based guidance about how to improve prognosis and QOL. There are almost no cohort studies that collect data on these factors.

In summary, the ROK study is one of the largest cohort studies investigating the effect of lifestyle, CAM use, and psychosocial factors on the prognosis of breast cancer. It includes one subcohort with blood and tissue sampling so that various hypotheses can be investigated. So far, the effect of these modifiable factors by patients is not known and this information is eagerly awaited by patients. The ROK study will yield important evidence in the near future.
